# Editorial: Advances in cutaneous microbial infections

**DOI:** 10.3389/fmed.2025.1573853

**Published:** 2025-02-26

**Authors:** Fuqiu Li, Liyan Xi, Sihong Song

**Affiliations:** ^1^Second Affiliated Hospital of Jilin University, Changchun, China; ^2^Department of Dermatology, Sun Yat-sen Memorial Hospital, Sun Yat-sen University, Guangzhou, China; ^3^Department of Pharmaceutics, University of Florida College of Pharmacy, Gainesville, FL, United States

**Keywords:** pathogenicity, virulence factors, host-microbe interaction, antimicrobial resistance, biofilm formation

Infectious diseases, caused by various microorganisms including bacteria, fungi, viruses, and parasites, have drawn increasing attention across various disciplines in recent years, influenced by factors such as the aging population and the growing use of immunosuppressive agents. As the largest organ of the human body, the skin is highly susceptible to a wide range of pathogens. In recent years, advances in the understanding, diagnosis, and treatment of these infections have transformed clinical practices, offering better outcomes for patients. Recent advances in understanding, diagnosing, and treating these infections have transformed clinical practices, improving patient outcomes. Molecular diagnostics, such as polymerase chain reaction (PCR) and next-generation sequencing (NGS), have enhanced the speed and accuracy of identifying pathogens. These techniques enable the detection of low-abundance or fastidious organisms that were previously challenging to diagnose. The growing challenge of antimicrobial resistance in managing cutaneous infections has spurred research into alternative therapies. Advances in topical and systemic antimicrobial agents, including liposomal formulations and combination therapies, have demonstrated improved efficacy and reduced side effects. Additionally, novel treatment strategies, such as antimicrobial peptides, are emerging as promising options for drug-resistant infections. This Research Topic aims to elucidate the epidemiological trends, distinct clinical manifestations, recent advances in pathogenesis, and the emergence of drug-resistant strains associated with infectious skin diseases. It seeks to provide guidance for monitoring drug-resistant infections and to propose targeted prevention and control measures, thereby informing clinical best practices. This Research Topic includes two research articles, one case report, and one review article, covering studies on skin diseases or pathogens related to bacterial, fungal, and viral infections, further enriching the knowledge base of infectious skin diseases.

Cutaneous granulomas are a heterogeneous group of diseases characterized by skin inflammatory responses caused by various stimuli, including infections, foreign bodies, malignancies, metabolites, and chemicals. However, virus-associated chronic cutaneous granulomas are uncommon and are often linked to immunodeficiency. Wang et al. reported a case of rubella virus-associated cutaneous granuloma in a patient presenting with recurrent, painless ulcerative and crusted plaques on the right lower limb. Auxiliary examinations ruled out the possibility of bacterial, fungal, and mycobacterial infections. Skin biopsy followed by metagenomic next-generation sequencing (mNGS) revealed rubella virus infection. Whole-exome sequencing of the patient identified a homozygous mutation in the TAP1 gene, which had not been previously reported as pathogenic. This novel finding holds potential significance for the diagnosis and research of immunodeficiency disorders.

The gut microbiota, as a vital ecosystem within the human body, has a significant impact on the host's immune system, metabolism, and nervous system. Recent studies have shown that gut microbiota imbalance is closely associated with the occurrence and progression of various diseases, including skin disorders. Meng et al. used bidirectional Mendelian randomization to analyze data from large-scale genome-wide association studies (GWAS) on gut microbiota and herpes zoster (HZ). They identified a potential bidirectional causal relationship between the gut microbiota and HZ. These findings could pave the way for novel therapeutic approaches to address the global burden of HZ.

As one of the most common subcutaneous fungal infections, sporotrichosis is transmitted zoonotically through traumatic inoculation of contaminated soil or plants, or through bites and scratches from infected animals, posing a global threat to both humans and animals. Despite its prevalence, treatment options for sporotrichosis remain limited. Li et al. innovatively investigated the antibacterial peptide In-58 for its *in vivo* and *in vitro* antifungal activity against *Sporothrix globosa*. They discovered that this peptide exhibits significant antifungal properties, highlighting its potential as a novel therapeutic agent for sporotrichosis. This study provides a theoretical foundation for the future clinical translation of antimicrobial peptides.

Leprosy is a chronic infectious disease caused by *Mycobacterium leprae*, characterized by its high disabling potential. Data show that Brazil has the second-highest incidence of leprosy in the world, following India, posing a significant challenge to public health. Brito Gonçalves et al. conducted a retrospective analysis of paucibacillary leprosy in Brazil based on research from the past 20 years. Their analysis revealed significant geographical heterogeneity in Brazilian paucibacillary cases, which has profound implications for subgroup analyses. Addressing these disparities through targeted interventions and localized analyses could enhance the effectiveness of leprosy control efforts in the country. Continuous monitoring of case classification and regional trends is essential for adapting strategies to effectively tackle this public health challenge.

In conclusion, the studies in this Research Topic highlight the need for large-scale interdisciplinary research to fully understand the complexities of various infectious skin diseases and their impact on public health. Further in-depth studies on different pathogens are essential to elucidate their mechanisms of infection and host immune responses, providing a foundation for the development of novel diagnostic and therapeutic approaches.

